# Temporal variability of sea surface temperature affects marine macrophytes range retractions as well as gradual warming

**DOI:** 10.1038/s41598-024-64745-7

**Published:** 2024-06-20

**Authors:** Rosa M. Chefaoui, Brezo D.-C. Martínez, Rosa M. Viejo

**Affiliations:** 1 Department of Biology and Geology, Physics and Inorganic Chemistry, Area of Biodiversity and Conservation, University Rey Juan Carlos (URJC), Móstoles, 28933 Madrid, Spain; 2 Global Change Research Institute (IICG-URJC), University Rey Juan Carlos, Móstoles, 28933 Madrid, Spain

**Keywords:** Ocean warming, SST long time series, Temperature variability, Atlantic macroalgae, Climate-driven extinctions, Seaweed range shifts, Ecology, Biogeography, Climate-change ecology

## Abstract

Record mean sea surface temperatures (SST) during the past decades and marine heatwaves have been identified as responsible for severe impacts on marine ecosystems, but the role of changes in the patterns of temporal variability under global warming has been much less studied. We compare descriptors of two time series of SST, encompassing extirpations (i.e. local extinctions) of six cold-temperate macroalgae species at their trailing range edge. We decompose the effects of gradual warming, extreme events and intrinsic variability (e.g. seasonality). We also relate the main factors determining macroalgae range shifts with their life cycles characteristics and thermal tolerance. We found extirpations of macroalgae were related to stretches of coast where autumn SST underwent warming, increased temperature seasonality, and decreased skewness over time. Regardless of the species, the persisting populations shared a common environmental domain, which was clearly differentiated from those experiencing local extinction. However, macroalgae species responded to temperature components in different ways, showing dissimilar resilience. Consideration of multiple thermal manifestations of climate change is needed to better understand local extinctions of habitat-forming species. Our study provides a framework for the incorporation of unused measures of environmental variability while analyzing the distributions of coastal species.

## Introduction

Ocean warming has been increasing at an unprecedented rate since the 1950s and this pattern is projected to continue in the future with uncertain consequences on centennial timescales^1^. In this context, range boundaries of marine species are changing^2,3^, and the community composition of marine ecosystems has been altered^4^. Given that ocean warming is not uniformly occurring across the oceans at a constant pace^1,5^, it is unclear in which direction species and communities will shift^6^ or if they will be able to cope with climate change through plasticity/adaptation strategies^7^. The patterns of fluctuation around average sea warming trends are also changing, with recent rises of extreme events. Marine heatwaves (MHWs) were longer and more frequent over the past century^8^ and this trend is projected to further increase^9,10^. MHWs have been identified as responsible for alterations in species distributions and biodiversity patterns^3,11^, mass mortalities^12^, biological changes at individual, population and community levels^13^, and various impacts on ecosystem services^14^. However, most of these effects have been observed in regions where MHWs frequently reach categories higher than “moderate” (category I according to Ref.^15^). Since species distribution predictions are mostly based on warming trends, the need for improved understanding of the implications of extreme events and long-term pervasive climate change has become imperative to predict their impacts on marine species^11,16^.

Much less is known about the influence of temporal patterns of temperature variability on species range shifts. Marine organisms regularly experience environmental variability, prompting questions about how these dynamics may affect species’ susceptibility to climate change. In disturbed ecosystems, a range of variability patterns in environmental factors may turn into stressors. For example, the temporal variance is known to interact with the mean intensity of climatic events to modify the diversity and structure of benthic assemblages^17,18^. As a result, there is a growing interest among aquatic ecologists in the effects of changes in the patterns of variability under climate warming, and research including descriptors for the dynamics and complexity of abiotic factors is rising^19,20^. Among the different measures of variability suitable to examine ecological changes, the environmental predictability, estimated by both the seasonality and the colour of environmental noise, has long been considered a driver of life-history evolution, population dynamics and biodiversity patterns (Refs.^21,22^ and references therein). Both components provide insights of different aspects of predictability, while the seasonality is associated to the regularity of fluctuations in data over seasons (i.e. a year; Ref.^22^) the environmental noise (i.e. environmental colour, colour of noise) estimates the temporal autocorrelation between successive values in the time series^23^. Temporal variability can be also assessed using common indices such as the coefficient of variation (CV), and the consecutive disparity index (D) that considers the chronological order of time series data^24^. Finally, changes in the skewness (the degree of asymmetry of probability distributions estimated from time series data) can be an effective early warning signal of ecosystem regime shifts^25^. However, the application of these metrics, including components of variability, has been scattered in ecological systems, and they have never been utilized jointly to evaluate their impact on the range shifts and local extinction of species.

Regarding how species are impacted by thermal fluctuations, responses vary widely among marine organisms, which have been observed to shift in phenology and/or acclimate, show tolerance, and accumulate positive or negative effects^20,26^. Species responses to same patterns of temporal variability and environmental events are probably modulated by species functional traits, e.g. life-history and ecophysiological responses. The relevance of using functional traits to investigate which marine species will be more vulnerable to different characteristics of MHWs has already been highlighted^27^. Species with distinct lifespan and generation times experience the same patterns of environmental fluctuations differently and so, their different “ecological memories” would influence their responses to warming^28^. Also, between-species variation in physiological thresholds may arise even in organisms that currently share similar geographic distributions, possibly because of evolutionary legacies^29^. To anticipate how projected ocean warming will affect marine ecosystems it is necessary to better understand the influence of species’ trait diversity in their responses to environmental changes.

At a rapidly changing environment, there are three distinct outcomes for populations: persistence through adaptation to new conditions at existing places, persistence through migration to other places tracking the same conditions, and extirpation (i.e. local extinction)^30^. Currently, the responses of marine canopy-forming species to thermal stressors are not fully understood and causal factors leading to local extinctions (i.e. extirpations) remain speculative. Habitat forming seaweeds (mostly species from the orders Fucales, Laminariales and Tilopteridales), provide the foundation of highly diverse and productive coastal ecosystems. These large brown algae, commonly named fucoids and kelps, form intertidal and subtidal forests in temperate and subpolar rocky reefs, being the dominant primary producers and supplying food and shelter for a diverse assemblage of associated species^31^. These marine macrophytes are globally in decline, particularly the populations at their warm trailing range edges^14^. There are well documented range contractions of native macroalgae, recognizing the crucial role played by the warming trend in coastal sea surface temperature (SST) and MHWs (e.g.^3,14,32–34^). However, there are still a lot of unanswered questions surrounding the SST components particularly responsible for the local extinction or persistence of macroalgae with similar worldwide distributions. Distinct responses of species may result from differences in their thermal thresholds and thus in their sensitiveness to the absolute magnitude of change, but sometimes the highest temperatures do not trigger the strongest biological impact on macroalgae, suggesting that species resilience is dependent on combinations between prolonged warming and short-term stress events^35^. Still, other components describing the variation in the long-term temporal distribution of SST (i.e. warming trends, SST predictability, etc.) and temporal changes in frequency, intensity, and duration of extreme events remain fairly unexplored for macroalgae. For example, marine cold spells (MCSs) are often understudied despite their impacts on marine ecosystems and foundational species^36^. Also, extreme events can have different impact depending on the preceding temperatures to each event (“preconditioning factors”; Ref.^15^), and on the biological processes related to the macroalgae life cycles at each season.

Here, we investigated the sea surface temperature-related factors that explain persistence and local extinction (i.e. extirpation) of six macroalgae species at their warm range edges, in the North of the Iberian Peninsula. In this region, there is a longitudinal SST gradient from cooler (West) to warmer (East) waters and the recent intertidal and subtidal rocky shore macroalgae retractions (mostly in the eastern coast) have been attributed primarily to changes in SST (e.g.^33,34,37–42^). We decomposed the effects of climate change on macrophytes extirpations using SST time series data corresponding to a baseline survey and a resurvey of macroalgae. To account with the previously mentioned factors, we analyzed the effects of: (i) annual and seasonal warming trends; (ii) annual and seasonal frequency, intensity, and duration of MHWs and MCSs; (iii) temporal variability measures; and (iv) species thermal thresholds for survival. This study seeks to determine which components of thermal temporal patterns are driving range shifts (local extirpations) and persistence of macroalgae along a longitudinal SST gradient at their warm range edges. We also determined whether every species is responding in the same way attending to its life cycle and thermal tolerance. This study aims to provide new insights into the understanding of the effects of climate change on macrophytes, with the incorporation of previously unused measures of thermal variability.

## Results

**Figure 1:**
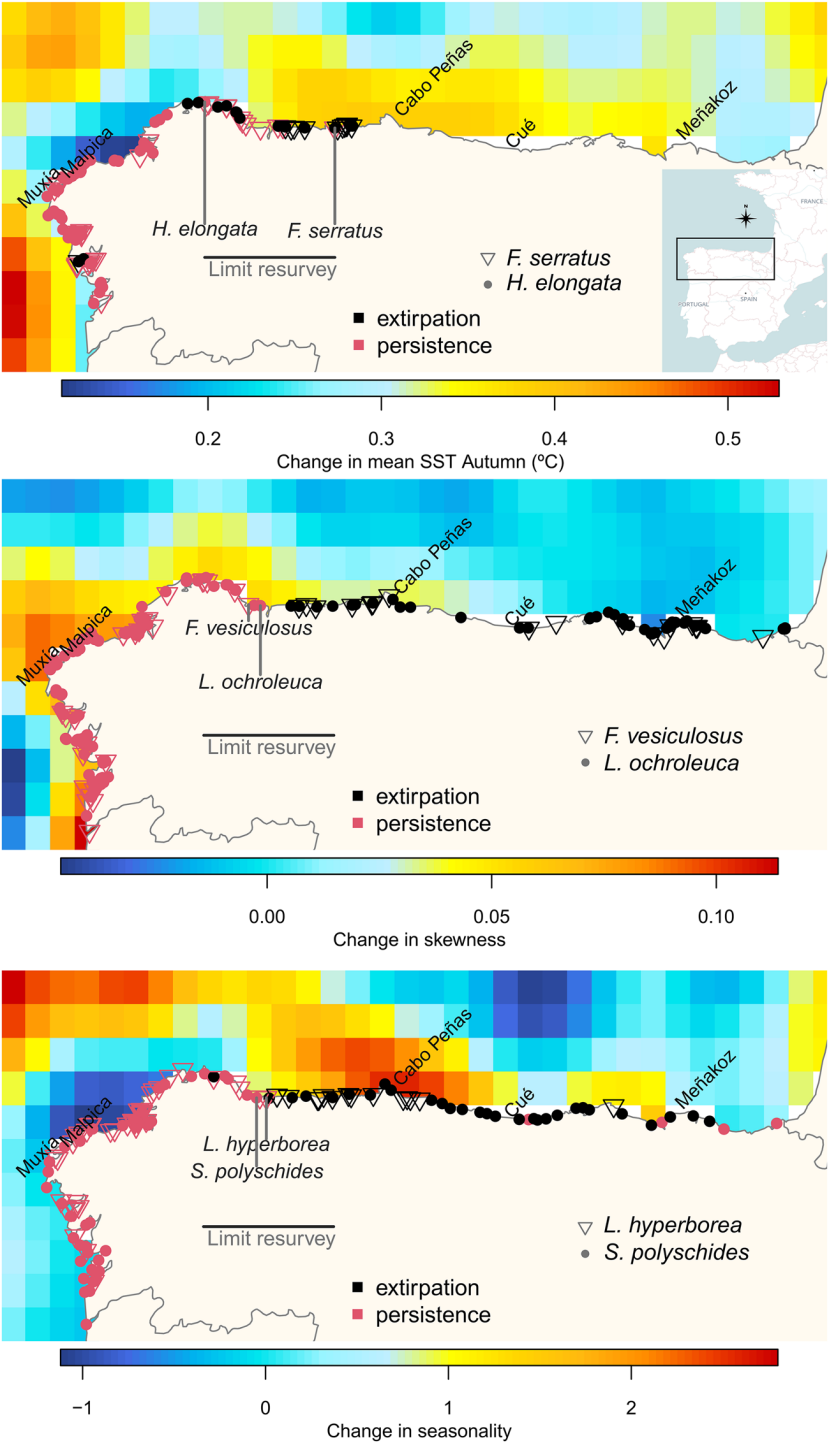
Persistence and local extinction (extirpation) of macroalgae populations between two surveyed periods along the Northern Spanish coast. Populations of *Fucus serratus, F. vesiculosus, Himanthalia elongata, Laminaria hyperborea*, *L. ochroleuca* and *Saccorhiza polyschides* were surveyed at two periods: baseline (1980–1990) and resurvey (2013–2015) and persistence/extirpation of populations analyzed using sea surface temperature series data. Distributional limits in the resurvey period are shown as vertical bars (for *S. polyschides*, there were still a few sparsely populations east of the given location). The change in the variables (mean sea surface temperature (SST) of autumn, seasonality, and skewness) represents the anomaly (difference) between the two periods.

**Figure 2:**
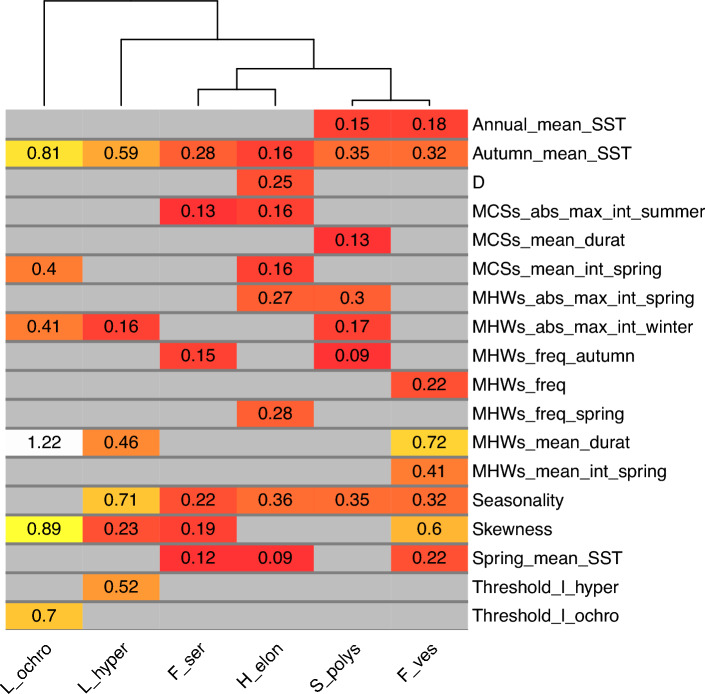
Most relevant variables discriminating between sites with persistent and extirpated populations for each macroalgae species. Heatmap shows the independent contribution (I) of variables obtaining an I value above the median (0.5 percentile) after the iterative hierarchical partitioning analysis. The light colors represent high values of I, while the dark colors represent low values. The column dendrogram shows clustering of species whose extirpation/persistence patterns are explained by similar factors. See Table S1 in Supporting Information for a description of the variables, and Fig. S1 in Supporting information for complete results also showing the variables with I values below the 0.5 percentile in the hierarchical partitioning analysis. *SST* sea surface temperature.

In the north of the Iberian Peninsula, the retraction of six cold-temperate brown algae species was examined during two time spans, the baseline and resurvey periods (Fig. 1). Depending on the species, 29 to 39 from the initial 85 variables were not correlated and used for the hierarchical partitioning analyses. After running the iterative hierarchical partitioning analyses, from 8 to 11 variables were used in the final hierarchical partitioning analysis computed for each species. The most relevant variables discriminating between persistence and extirpation for each macroalgae were considered to be those showing an independent contribution above the median (I > 0.5 percentile) in the final hierarchical partitioning analysis (Fig. 2; see Fig. S1 Supporting information for the complete results). Change in autumn mean SST was the only variable found relevant for all species. Two variables measuring the temporal variability of SST, change in seasonality and change in skewness, also showed a high I value for five and four species, respectively. The rest of variables discriminated differently between persistence and extirpation depending on the species. Many variables related to changes in MHWs and MCSs showed I values below the 0.5 percentile, with some exception, i.e. the MHWs mean duration (Fig. S1; Supporting information). The dendrogram showed *L. ochroleuca* and *L. hyperborea* were related to each other (i.e. their clusters were close; Fig. 2), suggesting extirpation/persistence patterns were explained by similar factors, which differed from those of other species. For example, the thermal thresholds were relevant discriminants only for *Laminaria* species (Fig. 2). Similarities were also found between *F. serratus* and *H. elongata*, with MCSs in summer being relevant for only those two species, and in another cluster formed by *S. polyschides* and *F. vesiculosus.*

**Figure 3:**
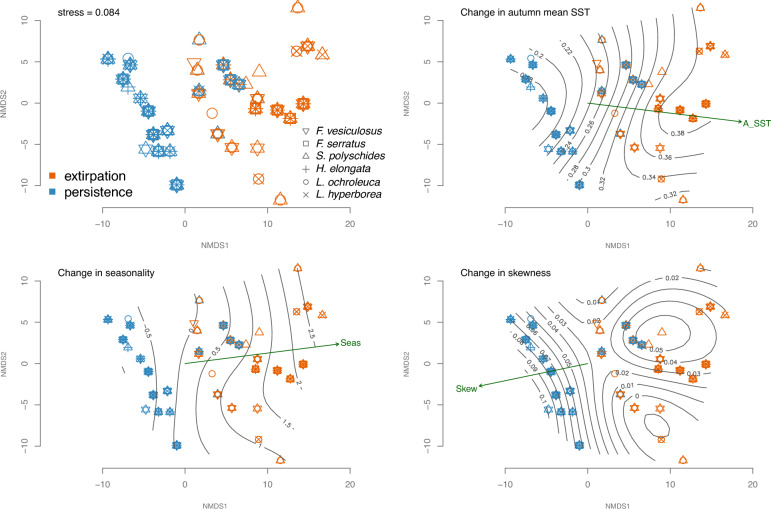
Non-metric multidimensional scaling (NMDS) ordination of the sites (i.e. locations, grids) where extirpation or persistence has occurred over the baseline and resurvey periods in the Northern Spanish coast. Smooth surfaces of the change in autumn mean sea surface temperature, seasonality and skewness fitted by means of generalized additive models showed a gradient direction able to explain the extirpation-persistence distribution (e.g. extirpated populations tend to increase when the seasonality anomaly increases over time). Macroalgae populations studied are *Fucus serratus, F. vesiculosus, Himanthalia elongata, Laminaria hyperborea*, *L. ochroleuca* and *Saccorhiza polyschides.* See Supporting information for a representation showing the vectors of all the variables used (Fig. S2), a description of the variables (Table S1), and the NMDS scores (Table S2). *SST* sea surface temperature.

**Figure 4:**
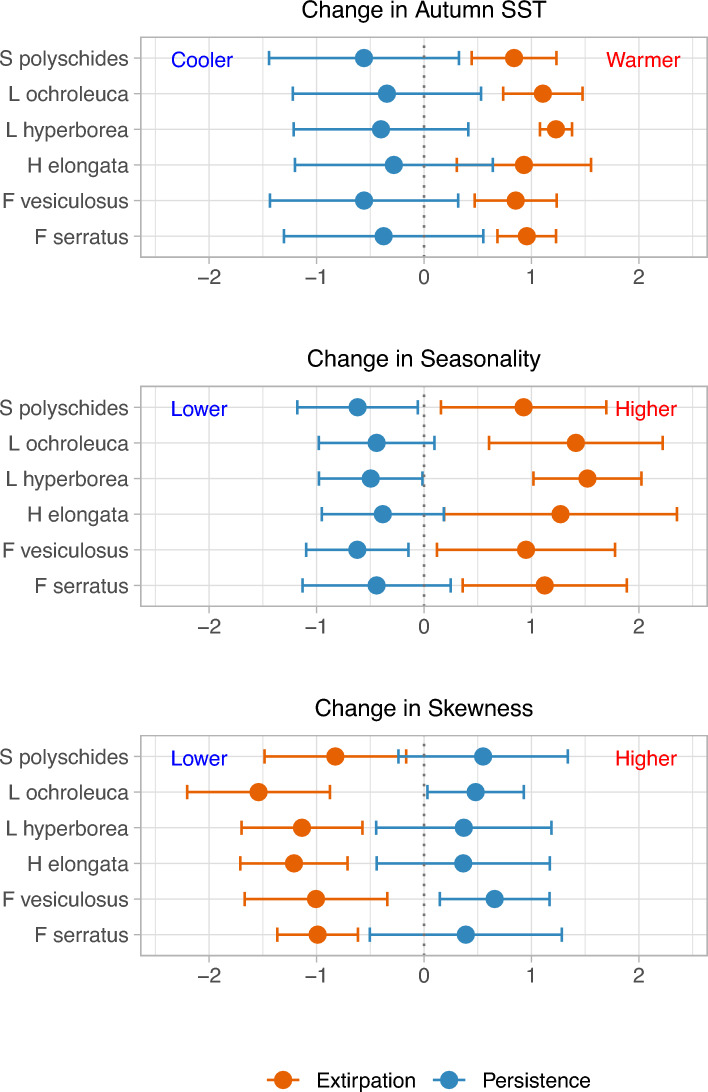
Change in autumn mean SST, seasonality and skewness between the baseline (1982–1990) and resurvey (1991–2015) periods at extirpated and persistent populations of macroalgae. Anomalies of variables were standardized into z-scores to provide a better comparison. For each species, circles indicate the mean z-score of all sites and error bars indicate standard errors. *SST* sea surface temperature.

The two-dimensional NMDS provided a reliable representation (stress value = 0.084; Fig. 3) of persistence and extirpation of macroalgae populations across the environmental space, defined by all the variables selected by the iterative process above mentioned and used in the final hierarchical partitioning analysis (Fig. S2; Supporting information). Axis 1 of the NMDS (NMDS1) discriminated persistent from extirpated populations of the six macroalgae species. Regions of extirpation were mainly related with greater temporal changes in seasonality and autumn mean SST, as well as with lower anomalies in skewness with respect to persistence grids (Figs. 3, 4, Table S2; Supporting information).

**Figure 5:**
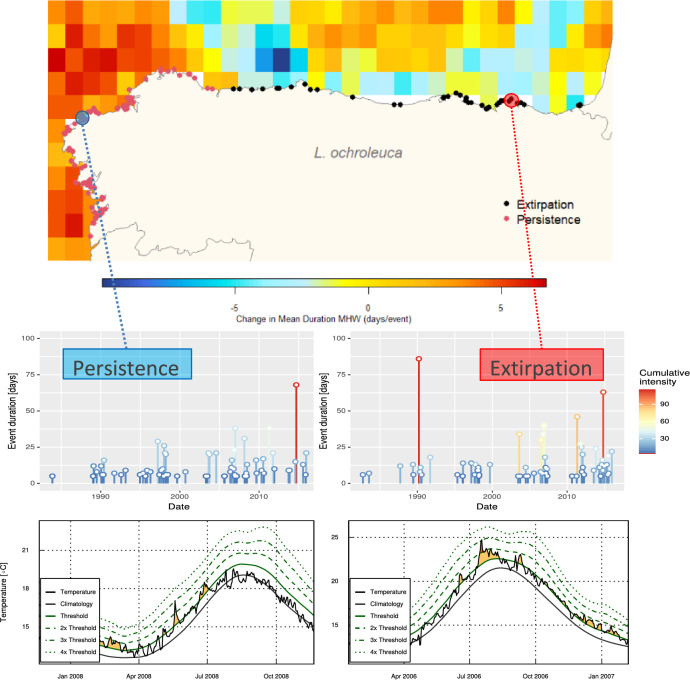
*Laminaria ochroleuca* populations exhibiting the most differing anomalies in mean duration (days/event) of marine heatwave (MHW) events. The most positive change between periods in MHW mean duration was found in persistent populations in La Coruña (Galicia; blue circle), where the MHW event with the highest cumulative intensity occurred in September 2014, and the event with maximum intensity in May 2008 (category II). On the other hand, negative anomalies in MHW mean duration were found in extirpated populations in Pais Vasco (red circle), which showed the MHW with highest cumulative intensity in March 1990, and the maximum intensity in September 2006 (category II). Height of the lolliplots represent the duration of the MHW event and the color shows the events’ cumulative intensity. Bottom panels illustrate the MHW with the highest values of maximum intensity detected from 1982 to 2015 in the persistence (left; max. intensity: 2.8683, date: 2008-05-19) and extirpation (right; max. intensity: 4.1367, date: 2006-07-18) regions.

Variables related to changes in extreme events (MHWs and MCSs) between the baseline and resurvey periods showed relevance discriminating persistence and extirpation in the hierarchical partitioning analyses of some species (Fig. 2, Fig. S1; Supporting information) and in the NMDS (Fig. 3, Table S2; Supporting information). Remarkably, extirpated populations showed negative anomalies (reductions) in mean duration of MHWs between the analyzed time periods, while most persistent populations had positive anomalies (increases, Fig. 5). MHWs frequency and intensity also showed reductions in extirpated populations (Figs. S2, S3; Supporting information). In general, MCSs showed less relevant relationships with extirpation/persistence patterns (Figs. 2, 3). The anomalies in MCSs were positive in areas with local extinctions while in persistence areas no changes or very reduced anomalies were detected (Fig. S3; Supporting information).

**Figure 6:**
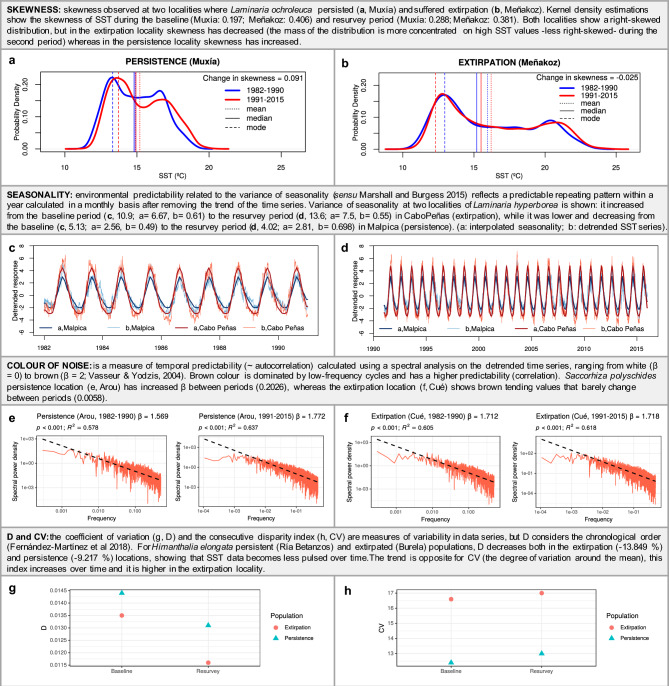
Performance comparison of variability metrics at locations where macroalgae persistence and local extirpation occurred between the baseline (1982–1990) and resurvey (1991–2015) periods. *SST* sea surface temperature.

In average, extirpated populations suffered a higher positive anomaly between periods in autumn mean SST (mean ± SD = 0.36 °C ± 0.017) than persistent populations (mean = 0.23 °C ± 0.03) (Fig. 4, Table S3; Supporting information). Overall, skewness was positive at the studied area over time (mean skewness baseline = 0.36 ± 0.08; mean skewness resurvey = 0.37 ± 0.07). However, extirpation regions experimented a lower change in skewness (mean = 0.023 ± 0.016) than persistent populations (mean = 0.067 ± 0.004) (Figs. 3, 4, 6, Fig. S3, Table S3; Supporting information). Anomaly in seasonality was positive in extirpated populations (mean = 1.39 ± 0.34) but slightly negative in persistence regions (mean = −0.29 ± 0.26) (Figs. 3, 4, 6, Fig. S3, Table S3; Supporting information). D index was only found relevant for *H. elongata* (Fig. 2). D is higher in the persistent populations, and it has been decreasing over time in all the study area, but particularly in the extirpation zone, indicating a reduction in disparity (i.e. SST data becomes less pulsed over time) (Fig. 6, Figs. S3, S4; Supporting information). We did not find the colour of environmental noise to be a relevant discriminant of persistence/extirpation.

## Discussion

Our study identified associations between the persistence and extirpation of macroalgae populations and variables related to gradual warming and temporal variability of sea surface temperature (SST). We found local disappearance of populations are associated to the temporal variability in SST, estimated with variables not typically considered in studies on how macroalgae are affected by climate change. Populations that suffered extirpation were in regions with greater SST warming in autumn (NE Spain, i.e. the inner Bay of Biscay), and have experienced an increase in seasonality (while most persistent populations showed a decrease), and a decrease in skewness over time (Figs. 3, 4). Species responses to environmental change may be complex and dependent on distinct aspects of a factor, involving not only its trend but also the variability^19,20,35^. Integrating historical occurrence data with two surveys at different periods allowed us to decompose the effects of SST on distributional shifts at the southern European range edge of these habitat forming species. The study area (Northern Spanish coast), with a SST gradient longitudinally oriented at a similar latitude, provides an ideal testing ground for a fine analysis of the effects of SST, as environmental changes presumably occur faster here than along a latitudinal gradient.

Autumn SST anomalies between the resurvey and historical periods were found to be a relevant factor explaining persistence/extirpation of all species (Figs. 1, 2). This season has been seldom considered when analyzing species distributions and the ecological impacts of climate change on marine macrophytes. Winter and/or summer sea temperatures are the commonly referred drivers of geographical boundaries of North Atlantic macroalgae by limiting growth and reproduction or by lethal effects^43–45^. However, autumn may be more important than previously assumed, in particular at the warming range margin of cold-temperate macroalgae. SST is increasing at higher rates in autumn than in other seasons in NW Iberia^46^ and autumn SST was found a relevant factor determining the fine-grain distribution of *F. serratus* in Atlantic rias of NW Spain^47^. Warmer autumns would extend the heat stress already experienced by seaweeds during the summer, with sublethal and lethal consequences in individuals and populations. Shorter reproductive periods, mainly in spring-early summer, and more variable reproductive output were detected in marginal populations of *Fucus* species in N Spain and Portugal^48–50^. The success of new macrorecruits in late summer-autumn may become critical for the persistence of fucoids with a short lifespan (2 to few years) such as *H. elongata* and *Fucus* spp., particularly if the reproductive window narrows. Furthermore, frond damage and weakening observed in adult plants of already extirpated populations of *F. serratus* in N Spain during autumn (authors, pers. observation) can cause individuals to be detached by storms more easily. In the annual kelp *S. polyschides*, sporophyte growth continues until autumn, when biomass in N Spain reaches its highest; afterwards spore release, germination and gametophyte formation occur^42,51^. Thus, prolonged, cumulative heat stress in autumn may reduce or collapse *S. polyschides* sporophyte growth and reproduction. Greater tissue damage in autumn-winter than in spring-summer was also detected in sporophytes of intertidal populations of a *Laminaria* species in British shores, suggesting chronic effects of high air and seawater temperatures during summer-autumn^52^.

Interestingly, range contractions of these seaweeds were also explained by anomalies in SST temporal variability, particularly by seasonality, and skewness. Seasonality (i.e. the predictability in timing and magnitude of seasonal fluctuations) was comparatively higher in regions with extirpations (eastern shores) than in regions of persistence (westernmost coast) both during baseline and resurvey periods. But the variance in seasonality increased even more across time where extirpations occurred, i.e. increasing the amplitude of seasonal SST fluctuations. This increase in seasonality implies higher predictability but also a greater exposure to thermal extremes and fluctuations that might have affected macroalgae populations (Figs. 3, 4, 6). On the other hand, persistence regions had lower seasonality also showing a tendency to become less seasonal over time (i.e. less regular and predictable), implying a smaller oscillation of SST which confers a temperature-buffered character to these localities. A major component of the NW Iberian Peninsula’s longitudinal SST gradient is the presence of intense spring-summer upwellings^53^. The lower seasonality in this region is probably related to the reduction in the amplitude of the SST seasonal cycle caused by the rise of cold and nutrient-rich water to the surface in spring-summer, as observed in this and other upwelling systems^54,55^. Further, the decrease in seasonality with time might suggest an intensification of the upwelling from 1982–1990 to 1991–2015 that could have favored the persistence of macroalgae populations in NW Iberian Peninsula. Nevertheless, the detected reduction in seasonality was small, and the present and future trends of the coastal upwelling in NW Iberian Peninsula are currently under discussion. Several studies have indicated a weakening of the upwelling in this region over the last decades (e.g.^56,57^) but not others^58^. According to Ref.^59^, future sea surface warming will enhance the stratification of the upper layers, diminishing upwelling and lowering the inflow of nutrients from the surface. Future monitoring should assess if upwelling trends could or not lead to further macroalgae extirpations. Regarding skewness, in the study area there is a general pattern in which SST is right skewed (positively skewed, i.e. with dominance of low SST values). Nevertheless, the persistence region showed an increase in skewness across time; while data are balanced towards higher SST values over the years (i.e. skewness decrease) in the extirpation locations. Hence, the increase in skewness in the NW Iberian Peninsula reflects a predominance of low SST values over time that benefited the persistence of large, brown canopy-forming algae of cold-temperate affinities in this region. Conversely, skewness reduction in the extirpation shorelines illustrates a prevailing pattern of rising SST, that could have contributed to the local extinctions of these six species. Changes in the asymmetry of time series data, quantified by changes in the skewness, have previously been referred to as an indicator of regime shifts, and together with other changes in variability can alert us to identify a reduction of resilience in the ecosystems^25^.

Despite pertaining to close taxonomic groups, the analyzed macroalgae, kelps and fucoids, showed different vulnerability to warming effects at their southern limits of distributions in the N Iberian Peninsula, evidencing distinct response to the temperature components (Fig. 2), which may be linked to variations in their traits such as lifespans or thermal tolerances. We found similarities in the SST components relevant for *F. serratus* and *H. elongata*, the two intertidal species that exhibited clear range shifts in the studied area. As explained above, both species have short lifespans (*H. elongata* is biennial and *F. serratus* life expectancy reaches up to 4–5 years in wave-sheltered places^60^). Their apparent sensitivity to environmental changes could be linked to these short life spans and their dependence on sexual reproduction, since adult plants of *F. serratus* also lack vegetative regeneration^61^. *F. vesiculosus* and *S. polyschides* are resilient species within fucoids and kelps, respectively, still showing disperse populations along the studied region, and both species seemed affected by the same SST factors. The higher resilience of *F. vesiculosus* relies in its higher thermal tolerance compared to other fucoids such as *F. serratus*^62^ and its ability to resprout from the holdfast^63^. The annual sporophytes of *S. polyschides* exhibit a greater ability to acclimatize to changing environmental conditions than those of perennial *Laminaria* species, in particular *L. ochroleuca,* which presented a similar geographic distribution^64^. Also, the bigger surface area of adhesion of the gametophyte of the annual kelp might allow to remain viable for more than one year, facilitating fast recruitment^65^. All these aspects might give *S. polyschides* lower susceptibility to warming and greater resilience than do the perennial *Laminaria* kelps. On the other hand, we found thermal thresholds were only relevant for the two studied perennial kelps (Fig. 2, Fig. S5 Supporting information). A recent study in Baja California also found a greater sensitivity of the giant kelp *Macrocystis pyrifera* to exceeding absolute thresholds than to relative changes in temperature^66^. The importance of the temporal thermal profile in determining responses of macroalgae, and the specificity of these responses have been recently acknowledged^35^. Further experimental studies are however necessary to disentangle the traits influencing specific responses.

Contrary to our expectations, changes in extreme events characteristics over time (both MHWs and MCSs) did not support causal inferences regarding persistence/extirpation. We did not find key metrics of extreme events explained the local extirpations, even examining their effect by seasons, as local extinctions occurred with negative anomalies (declines over time) in mean duration, frequency and intensity of MHWs (Fig. 5, Fig. S2; Supporting information). Despite the lack of explanatory capacity of MHWs in our study, we found the extirpation region was already affected by more frequent, intense and longer MHWs than the persistence area during the baseline period (1982–1990, Fig. 5). MHWs have been related to mass mortality events of marine benthic species in the Mediterranean Sea^12^, Australia^11,15^, see^13^ for a review. However, MHWs reached greater categories in these marine locations than in our study area, where only MHWs category II were recorded from 1982 to 2015. In our examined region, Smith et al.^13^ did not find notable MHW events compared to other temperate oceanic regions. So extreme heat events variation present in our study area could presumably not be sufficient to produce a measurable response in macroalgae. Besides, extreme events detection is known to be affected by the spatial and temporal resolution of SST data^15^ and shows differences with in situ datasets^67^. However, other authors^68^ using in situ nearshore SST time series data from 1998 to 2019 in two coastal areas in the middle Cantabrian Sea, were also unable to identify any significant trends in the frequency and duration of marine heatwaves. In general, the anomalies in marine cold spells (MCSs) were found less relevant discriminating the extirpation-persistence pattern of these populations close to their warmest latitudinal range edge, but further research might indicate if extremely cold water events could be a more relevant factor when analyzing extirpations at their poleward edges. Alongside from finding or not anomalies that could have caused local extinctions of macroalgae, our results raise concerns over the change in the extreme events regime in persistence coastlines. The increase in MHWs duration, frequency, and intensity in the persistence region (Galicia) could reflect an early sign of regime shift for the NW Iberian Peninsula, which has been a persistence region for macroalgae over decades.

This study set out to determine the effects of different components of temperature on macroalgae persistence-extirpation, since SST has been pointed out as the main responsible of seaweed retractions in the N Iberian Coast^33,34,37,39,40,42,69^, and worldwide (e.g.^11,14,32^). The relationship between variability of SST and the temporal patterning of occurrence under climate change is still understudied for macroalgae, and our research analyzing rarely used components of variability contributes to our understanding of local extinctions. Despite its exploratory nature, this study found strong associations between changes in SST components and the persistence and extirpation patterns. The persistent populations were benefited by less SST fluctuations and overall colder SST, whilst extirpated populations were affected by rising SST, with increases in autumn anomalies and seasonality. Although we did not find environmental noise to be a relevant explanatory factor, it is possible this variable might discriminate better within wider study areas, by covering all the variation that seawater can show, and covering a wider range of species with different life cycles and lifespan. Further research should be undertaken to investigate other factors not explored in this manuscript such as herbivorous pressure (e.g.^70^), the role of nutrient supply—a future nutrient depletion is linked to projections of upwelling weakening in the NW Iberian Peninsula^59,71^—wave action, water turbidity, or the impact of aerial stress in the case of intertidal species, which could also cause synergistic effects with SST. Also note that the biological response of the species can differ between populations and its recovery from stress depend on its continuity (e.g. the number of consecutive days that thermal thresholds were exceeded was not measured). Given expected increase in MHWs extremity with ocean warming^9,10^, it is important to continue monitoring its effect over time. Future monitoring should assess if the increase in this trend could lead to further macroalgae extirpations. Critical effects of temperature may be cumulative in time, and impacts may involve more than one life stage. Treatments applying a combination of sustained warming and MHWs produced different responses in forest-forming seaweeds^35^, suggesting the synergistic effects between different components also need further investigation. Temperature is a key factor for habitat-forming macrophytes and this work is a novel approach to assist in our understanding of the role of the different aspects of warming on distributional shifts.

## Methods

### Study area and biological data

The distribution of six cold-temperate brown algae species in the North of the Iberian Peninsula were compared between two time periods from 1980 to 2015: two species from the Laminariales order (*Laminaria hyperborea* and *Laminaria ochroleuca*)*,* one from Tilopteridales order (*Saccorhiza polyschides*), and three from the Fucales order (*Himanthalia elongata*, *Fucus serratus* and *Fucus vesiculosus*). These macroalgae have two southern boundaries in the Iberian Peninsula, one of them is in N Spain, and they are among the most relevant habitat-formers of the Northern Spanish coast subtidal and intertidal systems. Life cycles of kelps and fucoids clearly differ, since kelps have heteromorphic diplohaplontic cycles, with two alternate stages (the diploid macroscopic sporophyte and the microscopic gametophytes), while fucoids have monophasic life cycles. Lifespan also differ between the studied species. The fucoid *H. elongata* is biennial^72^ and *Fucus* spp. are perennial but with individuals living a few years (in *F. serratus* up to 5 years but only in sheltered places, see Rees^60^), while some *Laminaria* species, such as *L. hyperborea* have maximum ages around 15 years (see Schiel and Foster^31^ and references therein). The sporophyte of *S. polyschides* is annual, but in both annual and perennial species of kelps the microscopic gametophytes have unknown longevity^65^.

The studied area covered from the Miño River mouth in the south of Pontevedra (41° 52ʹ N, 8° 52ʹ W) to the France-Spain border (43° 22ʹ N, 1° 47ʹ W) (Fig. 1), a region affected by a longitudinal SST gradient from cooler (West) to warmer waters (East) (Fig. S6; Supporting information). We used a georeferenced database compiled from recent field surveys recording the presence of the species in the North coast of Spain, in combination with historical data obtained from the literature and technical reports (for all references, see Table 1 in Casado-Amezúa et al.^33^ review). The database used is openly available in Zenodo (see Data availability section). To assess the effect of seawater temperature on the distribution of the species, available information of presence/absence for all seaweeds was grouped into two time periods, following Casado-Amezúa et al.^33^: (i) the baseline period (1980–1990), and (ii) the resurvey in 2013–2015. These periods of biological data were examined using time series data. Since the daily OISST v2.1 data is only available from September 1, 1981 (see “Long term sea surface temperature data” section), we selected January 1, 1982 as the beginning of the SST series data to be analyzed. Thus, we examined the difference between the 1982–1990 SST time series data to describe the baseline period, and the 1991–2015 SST time series data to describe the resurvey period.

The same localities were consistently recorded in both periods at different times of the year during asynchronous visits for the different species. Intertidal species were surveyed at low tides and Laminariales and *S. polyschides* subtidal shallow-water populations (down to a maximal depth of about 20 m) by snorkelling and diving. The resurvey period represents a snapshot within an almost constant update of the species distributions in N Spain by the research group and a network of collaborators. For instance, in the case of *Fucus serratus* and *Himanthalia elongata* two field surveys were carried out in 2004–2006 and 2008–2009 by the research group^34,73^. All the targeted species gradually disappeared from the early 2000’s onwards in numerous localities of N Spain. Subsequent visits since 2015 to most of the locations confirm the local extinctions, except for *S. polyschides*, of which reduced populations have been recently found at some of the localities recorded as absences by 2015.

### Long term sea surface temperature data

Time series of daily sea surface temperature (SST) for the Northern Spanish coast were retrieved from 1982 to the end of 2015, encompassing the same time periods as the biological data sets. We used the NOAA 1/4° daily Optimum Interpolation SST data (daily OISST v2.1 data^74^) which is available from September 1981. We examined the difference between the 1982 and 1990 SST time series data to describe the baseline period of seaweed data, and the 1991–2015 SST time series data to describe the resurvey period in which persistence and local extinctions were detected. This long-term climate data combines observations from diverse platforms (buoys, satellites, ships and Argo floats) offering a high quality control (https://www.ncdc.noaa.gov/). Despite being the daily OISST data at a relative coarse resolution and showing limitations in near-coastal zone (e.g. pixels that are distant from the coast), it facilitates comparisons with other related studies on extreme events as it is a standard methodology and improves reproducibility at any geographical space. Besides, daily OISST data offers a high temporal resolution, needed to obtain the recommended 30 year time series data to estimate the climatology^15^.

To estimate long term warming, we calculated basic statistics from the daily OISST time series for each spatial grid point and time window (baseline and 2015 resurvey, henceforth). We measured the annual and seasonal mean of the SST time series for each pixel at the baseline (1982–1990) and 2015 resurvey (1991–2015). Seasons were related to the Northern Hemisphere, being summer June to August, and winter December to February. Daily SST data were retrieved using the “heatwaveR” package^75^.

### Extreme events detection and characterization

We detected extreme events using the SST data described above and following the consistent existing framework. Marine heatwaves (MHWs) are discrete prolonged anomalously warm water events and were detected according to the definition in Hobday et al.^15^, which establishes a period of 5 or more days above the 90th percentile threshold of the SST data. Both threshold and the baseline mean vary through the input time series, as they are both calculated within an 11-day window. MHWs are usually described by their frequency, intensity, and duration^11,14,15^. Furthermore, to test the relationship of trend changes in upwelling events with macroalgae, we examined marine cold-spells (MCSs). MCSs were detected following Schlegel et al.^36^ which determines the anomaly should persist for at least five days below the 10th percentile SST threshold. Both MHWs and MCSs and their derived metrics were identified using the “heatwaveR” package^75^. MHWs and MCSs were detected on the entire SST time series data. For each pixel, we detected extreme events (MHWs and MCSs) using the complete daily SST time series data (1982–2015—34 years) as an unique climatology period. Afterwards, we divided the information obtained into two time periods that coupled seaweed data surveys. Thus, we calculated a set of metrics describing the events of each period (1982–1990 and 1991–2015): the yearly frequency, the mean duration per event, the intensity (absolute maximum, mean maximum, and mean intensity), and the mean and maximum cumulative intensity (intended as the integral of intensity over the duration of the extreme event) (see^15^ for details). These metrics were estimated both as a mean value per year and seasonally for each period.

### Sea surface temperature variability and skewness

We calculated variability measures from the daily OISST time series for each spatial grid point and time period. We estimated two components of environmental predictability in each time window and grid cell: the predictability of seasonal trend and the colour of environmental noise^21,22^ using the “envPred” R package^76^. The predictability of seasonal trend, related to the concept of seasonality *sensu* Marshall and Burguess^22^, reflects the variance in time series due to the predictable seasonal pattern within a year. The seasonal fluctuations in data (“seasonality”), determined as the regularity in the periodicity and variance in the average environmental state over seasons (i.e. a predictable repeating pattern within a year), is associated to the predictability of the mean environmental state^22^. We computed the “unbounded” seasonality (a/b), which is the ratio between the variance of the seasonal trend (“a”) and the variance of the time series once the linear trends are removed (residuals, “b”)^76^. The seasonal trend (a) is the monthly average of SST across the time series, interpolated afterwards to reproduce the seasonal time series at the same timescale of the original data (days, in this case). The colour of environmental noise measures the predictability between consecutive points in the time series, ranging from predictable or temporal autocorrelated environments (“brown and red noise”) to those experiencing continuous changes (“white noise”)^23^. Marine environments are considered as some of the most “reddened” (i.e. low-frequency cycled) systems^23^. To estimate the colour of noise, a spectral analysis is performed on the detrended time series^22^. The linear regression of the natural log of spectral density as a function of the natural log of frequency is estimated. The resultant negative slope (β) indicates the colour of noise from white (0 ≤ β ≤ 0.5), red (0.5 < β ≤ 1.5) to brown (1.5 < β ≤ 2)^23^. Brown noise corresponds to a time series dominated by low-frequency cycles, having then a higher predictability (correlation).

Additionally, we estimated the variation of SST for each period. We used the coefficient of variation (CV) of SST for each period (CV = standard deviation × mean^−1^ × 100) as a measure of the variability or dispersion of SST in relation to the mean. A greater CV would indicate a higher variability of SST. This is the most common index for assessing variability, though it has some limitations. For instance, CV is dependent on the mean value and the length of the time series and is sensitive to very rare, extreme values, typical of skewed distributions. This is why we also used the consecutive disparity index (D), which assesses the consecutive variations in SST as an indicator of temporal variability. D is considered a more suitable metric of temporal variability for ecological studies in comparison with other indices as it is not sensitive to the mean and length of time series. Furthermore, it considers the chronological order of the time series data and thus its autocorrelation structure^24^. It has been used, for example, to measure the average rate of change of soil moisture due to varied precipitation treatments^77^. D produces a logarithmic proportional comparison of consecutive values (i.e. daily SST). We calculated D index for each temporal block and grid cell following Fernández‐Martínez et al.^24^:

$$D=\frac{1}{\left(n-1\right)}\sum \limits_{i=1}^{n-1}\left|\ln\frac{{p}_{i+1}}{{p}_{i}}\right|,$$

where p_i_ is the series value at time i, and n is the series length. D value ranges from 0 to infinite, and in our case, a higher consecutive disparity (D value) would indicate a maintained pulsed nature of daily SST over time.

Finally, we used the skewness as a measure of asymmetrical distribution of SST series around its mean value. A time series with zero skewness would show a symmetric distribution of the values around the mean. In our case, a positively skewed SST time series would show a predominance of small SST values respect to the mean (right skewed; a right tail in the density distribution), while negative skewed time series would indicate a dominance of large SST values. The skewness (to test if SST was positively or negatively skewed) was calculated using the TSA package^78^ in R.

### Thermal thresholds

For each time interval, we calculated the number of days per year exceeding the species thermal limit. Thermal thresholds were retrieved from the literature and corresponded to physiological experiments. If lethal maximum temperature differed between studies, we chose the most conservative value (i.e. the lowest temperature). Thus, we used 25 °C as the threshold for *F. serratus*^62^, 18 °C for *H. elongata*^79^, 23 °C for *L. ochroleuca*^80^*,* 24 °C for *S. polyschides*^81,82^, and 20 °C for *L. hyperborea*^62,80^ (Fig. S5 Supporting information). The thermal threshold of *F. vesiculosus* (28 °C^62^) was above the maximum SST registered in the time series (25.3 °C) and could not be integrated in the analyses.

### Statistical analyses

We analyzed the persistence and extirpations of the macroalgae species in each site across time (baseline and resurvey periods). To do that, observations on the populations were categorized as “one (1)” if the species persisted over the two periods of time (or was observed only during the second period; just a few cases), and “zero (0)” if the species was present in the first period but absent in the second time period (we also regarded it as zero for *F. vesiculosus* if it has virtually vanished from semi-exposed shores and was only found in very small populations in wave sheltered locations).

To reflect the change in climate conditions over time, we calculated the difference (anomaly) between the resurvey period (1991–2015) and the baseline period (1982–1990) for all independent variables related to average trends, extreme events and temporal variability of SST (Table S1; Supporting information). In the case of the consecutive disparity index (D), we estimated the percentage of change between periods. Thus, a decrease in D (i.e. a decrease in the disparity of SST) from one period to the next is reflected as a negative percentage change.

The complete set of anomalies (85 variables) were initially assessed using a Pearson correlation test to discard high positive correlations in all cells of the study area (r ≥ 0.70, p < 0.001; i.e. variables showing a very similar longitudinal pattern of change). Since many variables derived from extreme events (MHWs, MCSs) were correlated to each other, in these cases we performed an additional correlation analysis to discard the least significant variables according to our species distributions. We first excluded the extreme events derived anomalies with the highest variance inflation factor (VIF). Then, we tested their correlations only in the cells including species data to exclude the one of each correlated pair with the largest AIC (Akaike’s Information Criterion) in a bivariate model (“binomial” family) performed for each species. This analysis was performed using the “corSelect” function of the “fuzzySim” R package^83^. From 18 to 29 extreme events related variables were retained for each species for subsequent analyses.

We used hierarchical partitioning analysis to determine how the change of each variable relates to the distributional shifts of macroalgae between periods. This analysis determines the relative importance of each independent variable to each species^84^. Hierarchical partitioning uses all regression models in a hierarchy to identify the explanatory variables more independently correlated to the dependent variables, thus producing better deductions on the most likely causal factors than regressions^85^. We estimated the hierarchical partitioning algorithm *sensu* Chevan and Sutherland^84^ using the “hier.part” R package^86^. The independent contribution (I) of each variable to the pattern of persistence/local extinction for each species was estimated using a randomized routine of 100 repetitions specifying the binomial family in the function “rand.hp” and assessing the significance of the analysis using the upper 0.95 confidence limit. Because the maximum number of variables that can be analyzed in each partition is 12^86^, we used hierarchical partitioning analysis in an iterative process for each species. Since the variables seasonally characterizing extreme events were very numerous, we first used hierarchical partitioning to select the most relevant variables (with the highest I, up to 12) per species and type of extreme event (MHW, MCS). Afterwards, the statistically significant variables with I ≥ 0.75 quantile related to each type of seasonal extreme events were selected to be analyzed together with the rest of the variables in the final hierarchical partitioning analysis per species. Hierarchical partitioning results were represented generating a heat map and calculating a hierarchical clustering displayed as a dendrogram using the function heatmap.2 of the R package “gplots”^87^.

We further explored the overall relationship between persistence/extirpation and the variables selected by the previous hierarchical partitioning analyses, using a non-metric multidimensional scaling (NMDS) ordination. We computed a two-dimensional NMDS using 100 random iterations and Euclidean distances to calculate the dissimilarity index among sites (i.e. locations, grids) with persistent/extirpated populations of each species. We calculated the dissimilarity among sites with observed persistence and/or extirpation of seaweed species based on the change in SST metrics between time periods. We fit the vectors of the environmental variables to the ordination setting 999 permutations. A generalized additive model was also used to assess how well the variables fit on a smooth surface within the NMDS. We performed the analyses with the functions metaMDS, ordisurf and envfit of the R package “vegan”^88^.

### Supplementary Information

Supplementary Information.

## Data Availability

The data that support the findings of this study are openly available in Zenodo at 10.5281/zenodo.8414685, reference number md5:a07a87983a73f1ccdef85417ec652b8a.
