# Supplementary material for: Temporal variability of sea surface temperature affects marine macrophytes range retractions as well as gradual warming
Source: Sci Rep. 2024 Jun 20;14:14206. doi: 10.1038/s41598-024-64745-7 (PMC11190259; doi:10.1038/s41598-024-64745-7)

Supplementary information

Tables and Figures

Temporal variability of sea surface temperature affects marine macrophytes range retractions as well as gradual warming

Rosa M. Chefaoui[1,2*], Brezo D-C. Martínez[1,2], Rosa M. Viejo[1,2]

1 Area of Biodiversity and Conservation, Department of Biology and Geology, Physics and Inorganic Chemistry, University Rey Juan Carlos (URJC), Móstoles 28933, Madrid, Spain.

2. Global Change Research Institute (IICG-URJC), University Rey Juan Carlos, Móstoles 28933, Madrid, Spain

Contact Information:

*Rosa M. Chefaoui

e-mail: rosa.chefaoui@urjc.es

Table S1 List of variables initially used to calculate the anomalies between the resurvey period (1991-2015) and the baseline period (1982-1990) in the statistical analyses. All variables were calculated from time series of daily sea surface temperature (daily OISST v2.1 data; Huang et al 2021). (*) Variables in which both their annual and seasonal mean values (summer, autumn, winter, spring) were calculated (5 values per variable, so that the total number of variables were 85).

**Variables of change**	**Definition**	**Unit**
Mean_SST (*)	Mean sea surface temperature	ºC
CV	Coefficient of variation	unitless
D	Consecutive disparity index	unitless
Env_col	Colour of environmental noise	unitless
MCSs_abs_max_int (*)	Absolute maximum intensity of marine cold spells	ºC
MCSs_freq (*)	Annual frequency of marine cold spells	events/year
MCSs_max_cum_int (*)	Maximum cumulative intensity of marine cold spells	ºC days
MCSs_mean_cum_int (*)	Mean cumulative intensity of marine cold spells	ºC days
MCSs_mean_durat (*)	Mean duration of marine cold spells	days
MCSs_mean_int (*)	Mean intensity of marine cold spells	ºC
MCSs_mean_max_int (*)	Mean maximum intensity of marine cold spells	ºC
MHWs_abs_max_int (*)	Absolute maximum intensity of marine heatwaves	ºC
MHWs_freq (*)	Annual frequency of marine heatwaves	events/year
MHWs_max_cum_int (*)	Maximum cumulative intensity of marine heatwaves	ºC days
MHWs_mean_cum_int (*)	Mean cumulative intensity of marine heatwaves	ºC days
MHWs_mean_durat (*)	Mean duration of marine heatwaves	days
MHWs_mean_int (*)	Mean intensity of marine heatwaves	ºC
MHWs_mean_max_int (*)	Mean maximum intensity of marine heatwaves	ºC
Seasonality	Predictability of seasonal trend	unitless
Skewness	Asymmetrical distribution of sea surface temperature	unitless
Threshold_h_elon	Time over *Himanthalia elongata* thermal limit	days/year
Threshold_f_ser	Time over *Fucus serratus* thermal limit	days/year
Threshold_l_hyper	Time over *Laminaria hyperborea* thermal limit	days/year
Threshold_l_ochro	Time over *Laminaria ochroleuca* thermal limit	days/year
Threshold_s_polys	Time over *Saccorhiza polyschides* thermal limit	days/year

Table S2 Correlations (r^2 and p-values) and scores of environmental variables in the non-metric multidimensional scaling (NMDS). In bold are shown the variables obtaining also an independent contribution (I value) above 0.5 in the hierarchical partitioning analysis (see Fig. 2 in main text and Fig. S1 in Supporting information).

	r^2	*p*	NMDS1	NMDS2
**Annual_mean_SST**	0.37	0.001	0.5	0.35
**Autumn_mean_SST**	0.58	0.001	0.76	-0.09
**Seasonality**	0.87	0.001	0.92	0.12
**Skewness**	0.37	0.001	-0.6	-0.12
**Spring_mean_SST**	0.26	0.001	-0.5	-0.11
MCSs_abs_max_int_spring	0.07	0.001	0.26	0.08
MCSs_max_cum_int_winter	0.15	0.001	-0.2	-0.32
MCSs_mean_int_winter	0.14	0.001	0.1	0.36
**MHWs_freq**	0.25	0.001	0.05	0.49
**MHWs_mean_durat**	0.67	0.001	-0.69	-0.45
**MHWs_mean_int_spring**	0.16	0.001	-0.33	-0.23
**MCSs_abs_max_int_summer**	0.24	0.001	0.46	-0.17
MCSs_max_cum_int_summer	0.24	0.001	0.3	-0.39
**MHWs_freq_autumn**	0.51	0.001	0.45	0.55
MHWs_mean_durat_autumn	0.92	0.001	0.4	-0.87
**Threshold_I_hyper**	0.96	0.001	0.89	0.4
MCSs_mean_durat_summer	0.12	0.001	0.16	0.31
**MHWs_abs_max_int_winter**	0.37	0.001	-0.61	0.06
**Threshold_I_ochro**	0.02	0.029	0.11	0.11
MCSs_mean_int_autumn	0.17	0.001	-0.12	-0.4
**MCSs_mean_int_spring**	0.49	0.001	0.49	0.5
MHWs_abs_max_int_summer	0.05	0.002	0.1	0.19
**D**	0.25	0.001	-0.47	-0.15
MCSs_mean_max_int	0.23	0.001	0.46	-0.13
**MHWs_abs_max_int_spring**	0.73	0.001	-0.81	-0.26
**MHWs_freq_spring**	0.74	0.001	-0.85	-0.1
MHWs_mean_durat_summer	0.62	0.001	-0.78	0.09
MCSs_mean_cum_int_winter	0.24	0.001	0.48	0.05
**MCSs_mean_durat**	0.14	0.001	0.13	0.34
MHWs_freq_summer	0.01	0.165	0.1	0.06

Table S3 Overall anomalies at the surveyed persistent and extirpated populations of macroalgae. Anomalies were calculated as the difference between the resurvey period (1991-2015) and the baseline period (1982-1990) for all variables.

	Persistence			Extirpation		
	**Mean SST Autumn (ºC)**					
	Min.	Mean	Max.	Min.	Mean	Max.
S. polyschides	0.11	0.24	0.36	0.27	0.36	0.39
L. ochroleuca	0.11	0.22	0.35	0.27	0.35	0.39
L. hyperborea	0.11	0.2	0.35	0.34	0.38	0.39
H. elongata	0.11	0.23	0.35	0.25	0.33	0.39
F. vesiculosus	0.11	0.23	0.35	0.28	0.35	0.39
F. serratus	0.11	0.29	0.39	0.35	0.37	0.39
	**Seasonality**					
	Min.	Mean	Max.	Min.	Mean	Max.
S. polyschides	-1.12	-0.21	1.51	0.01	1.35	2.58
L. ochroleuca	-1.12	-0.43	0.85	0.08	1.25	2.62
L. hyperborea	-1.12	-0.53	0.85	1.14	1.92	2.62
H. elongata	-1.12	-0.45	0.06	-0.01	0.92	2.31
F. vesiculosus	-1.06	-0.31	0.85	0.14	1.32	2.58
F. serratus	-0.95	0.18	1.9	0.07	1.6	2.31
	**Skewness**					
	Min.	Mean	Max.	Min.	Mean	Max.
S. polyschides	-0.03	0.06	0.11	-0.03	0.02	0.05
L. ochroleuca	0.03	0.07	0.09	-0.03	0	0.05
L. hyperborea	0.03	0.07	0.09	-0.01	0.03	0.05
H. elongata	0.03	0.07	0.09	0.03	0.04	0.05
F. vesiculosus	0.03	0.07	0.11	-0.03	0.01	0.04
F. serratus	0.03	0.06	0.09	0.03	0.04	0.05

Figure S1

Independent contribution (I value) in the hierarchical partitioning analysis of variables discriminating between persistence and extirpation populations for each macroalgae species. Dendrogram at top shows clustering of species whose extirpation/persistence patterns can be explained by the same factors. See Table S1 in Supporting information for a description of the variables. SST: sea surface temperature.

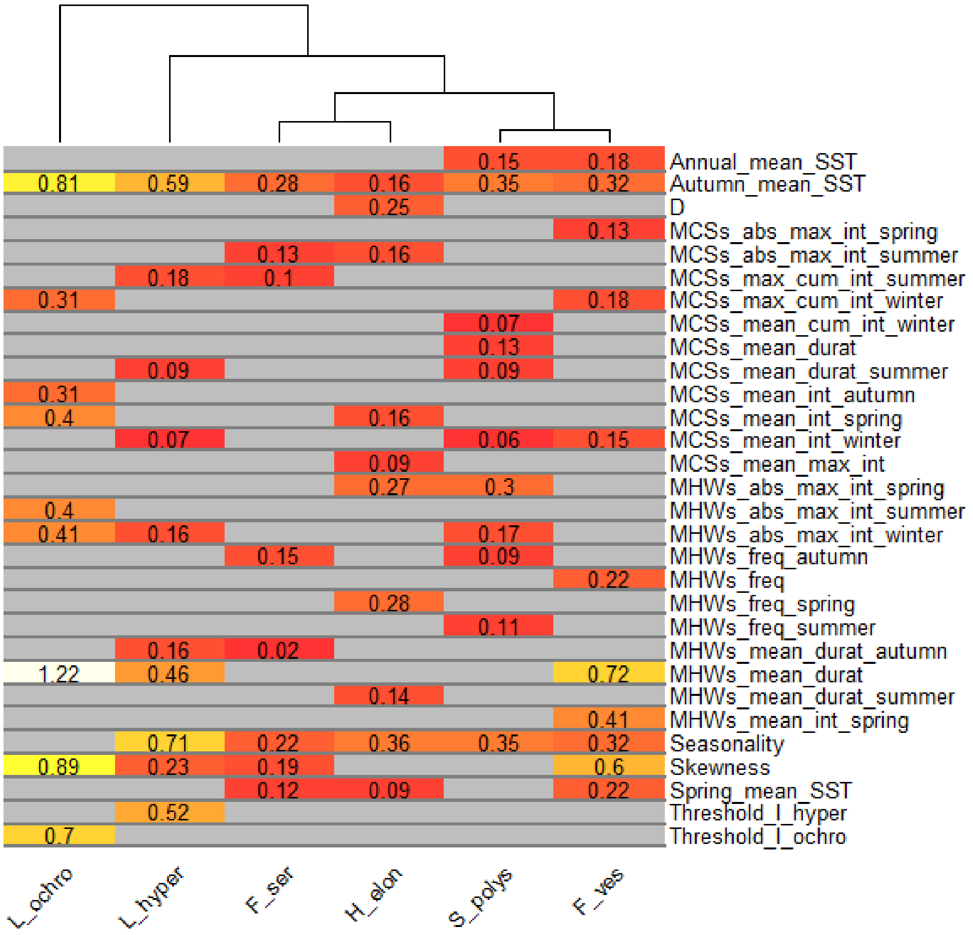

Figure S2

Non-metric multidimensional scaling (NMDS) ordination of the sites where extirpation or persistence has occurred over the baseline and resurvey periods in the Northern Spanish coast. The change in autumn mean sea surface temperature, seasonality and skewness showed a gradient direction able to explain the extirpation-persistence distribution (e.g. extirpated populations tend to increase when the seasonality anomaly increases over time). Longer arrows show stronger associations (r^2). Macroalgae populations studied are *Fucus serratus, F. vesiculosus, Himanthalia elongata, Laminaria hyperborea*, *L. ochroleuca* and *Saccorhiza polyschides.* See Supporting information for a description of the variables (Table S1), and the NMDS scores (Table S2). SST: sea surface temperature.

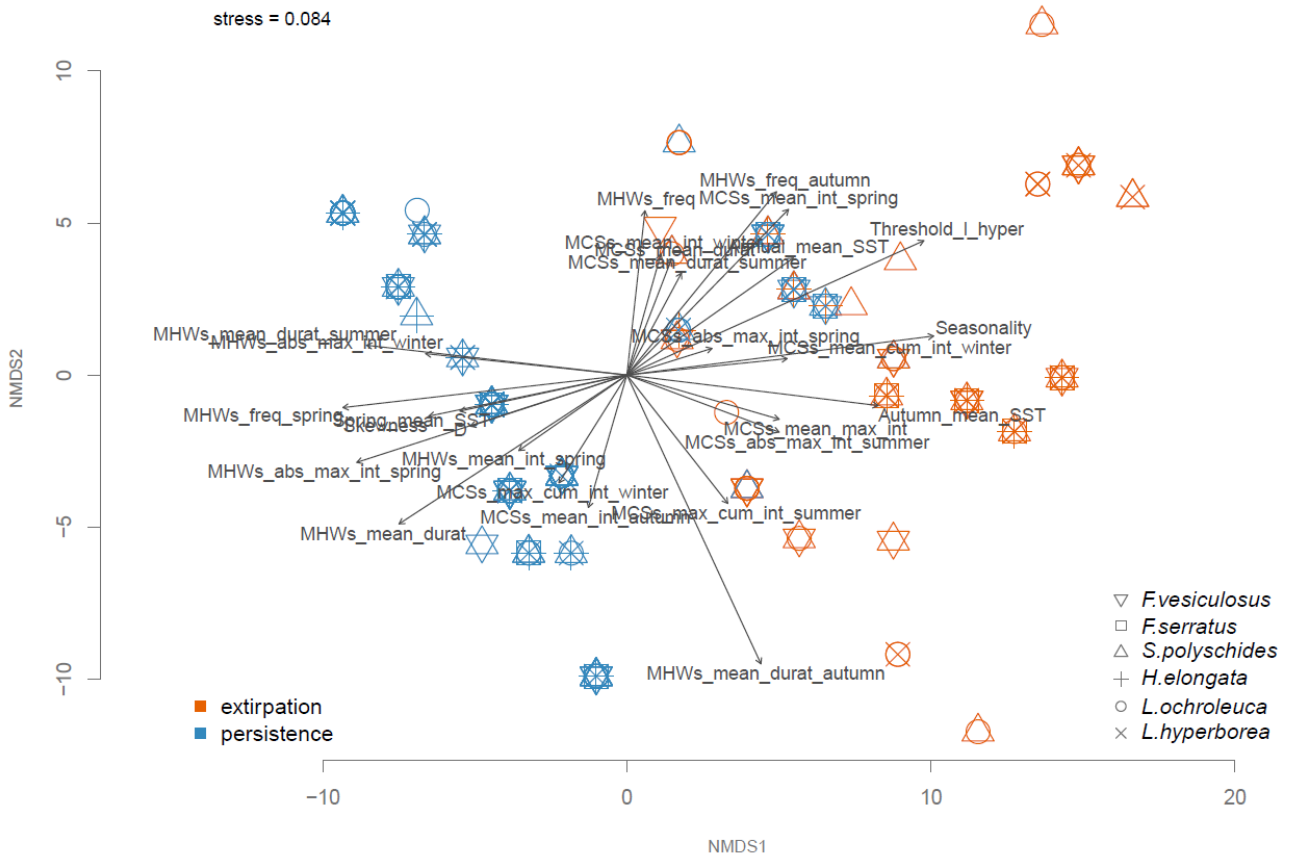

Figure S3

Examples of anomalies in variables related to marine heatwaves (MHWs) and marine cold spells (MCSs) at extirpated and persistent populations of macroalgae (black and red dots respectively). Anomalies were calculated as the difference between the resurvey period (1991-2015) and the baseline period (1982-1990) for all variables.

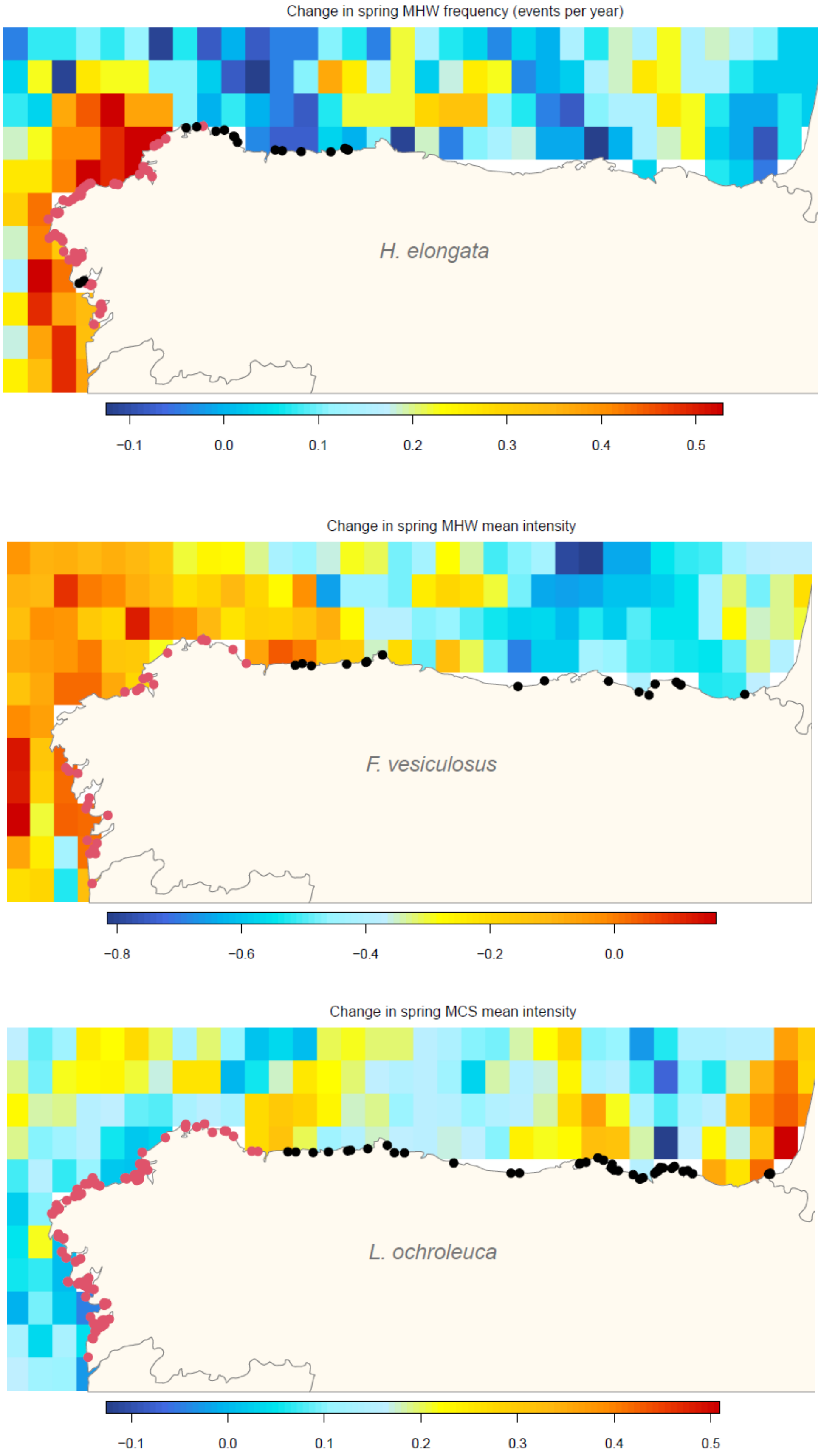

Figure S4

Performance of the consecutive disparity index (D; Fernández-Martínez et al 2018), a measure of **temporal variability**. Here we provide a comparison of a real sequence of sea surface temperature (SST) and a randomly generated time series with similar longitude, minimum and maximum values. The real data (in red) is a one-year time sample sequence SST from Burela (Galicia), where local extirpation of *Himanthalia elongata* occurred. D is higher in the random series due to its lack of autocorrelation between consecutive data (greater fluctuations) in comparison with the real SST time series.

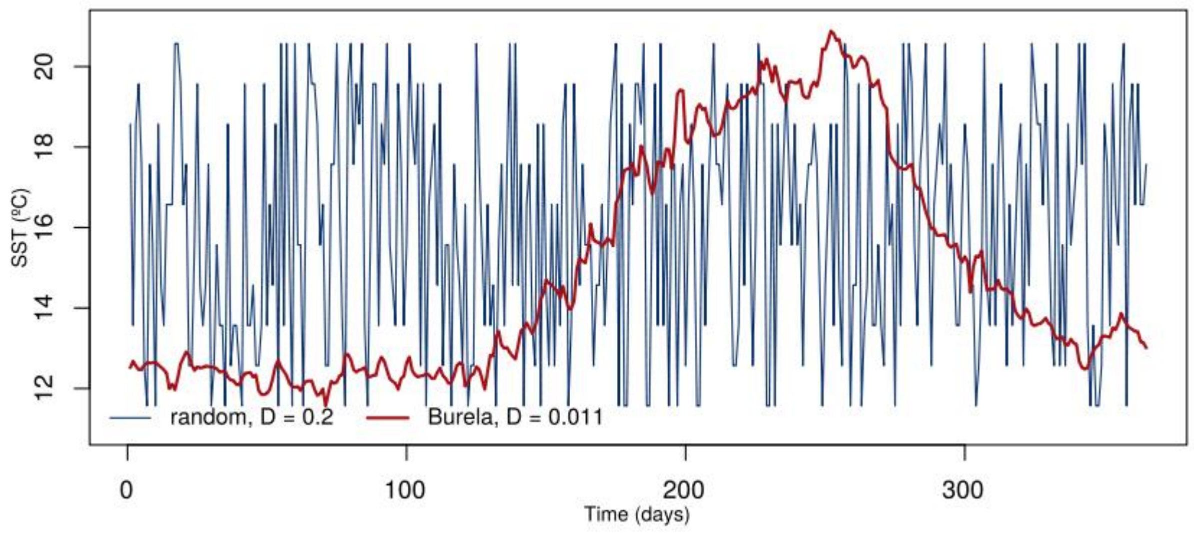

Figure S5

Anomalies in the frequency of days above the specific thermal threshold of each macroalgae species at extirpated and persistent populations (black and red dots respectively). Anomalies were calculated as the difference between the resurvey period (1991-2015) and the baseline period (1982-1990). The anomalies for *Fucus vesiculosus* are not shown since its thermal threshold was above the maximum SST registered in the time series.

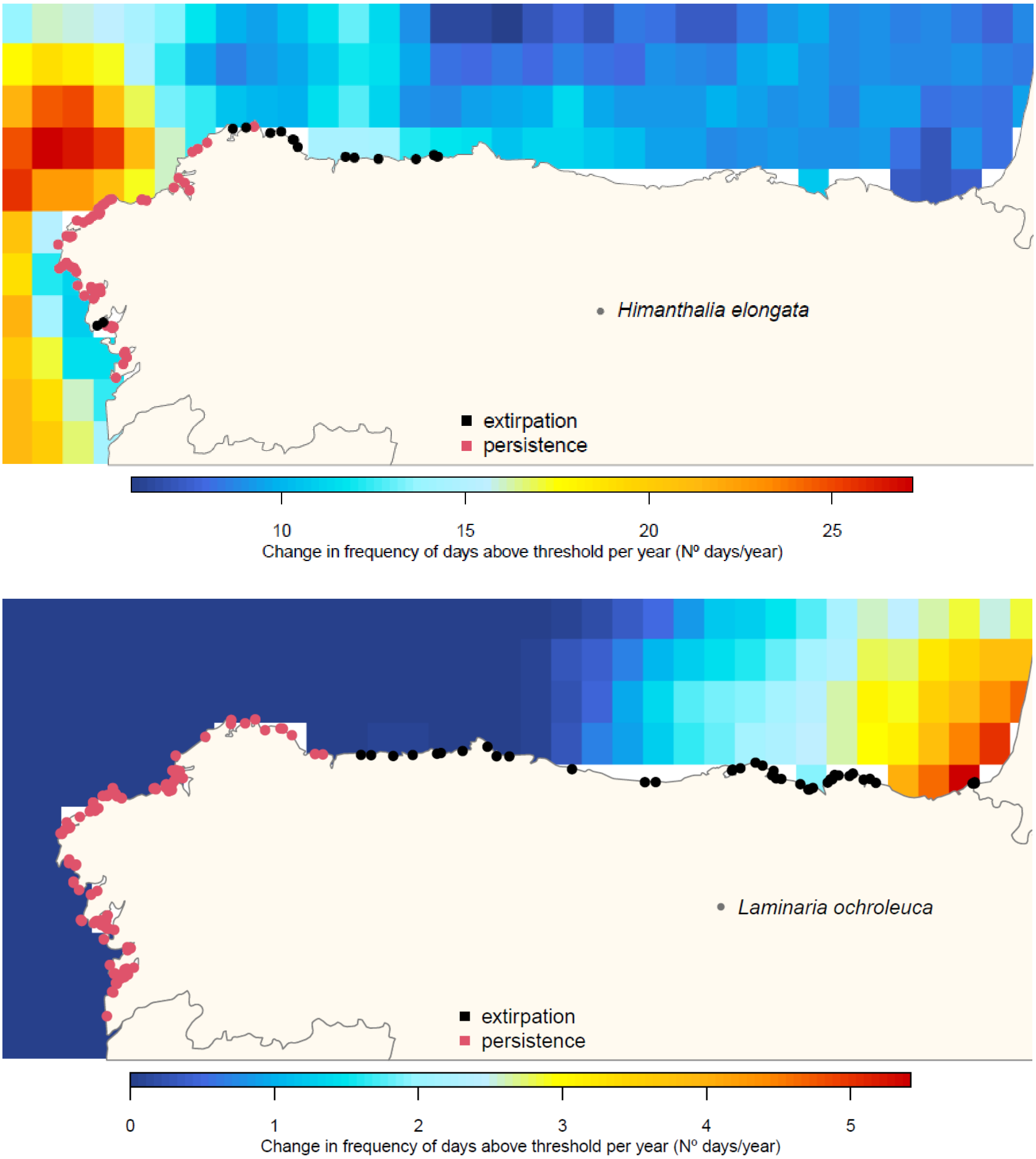

Figure S5 (cont.)

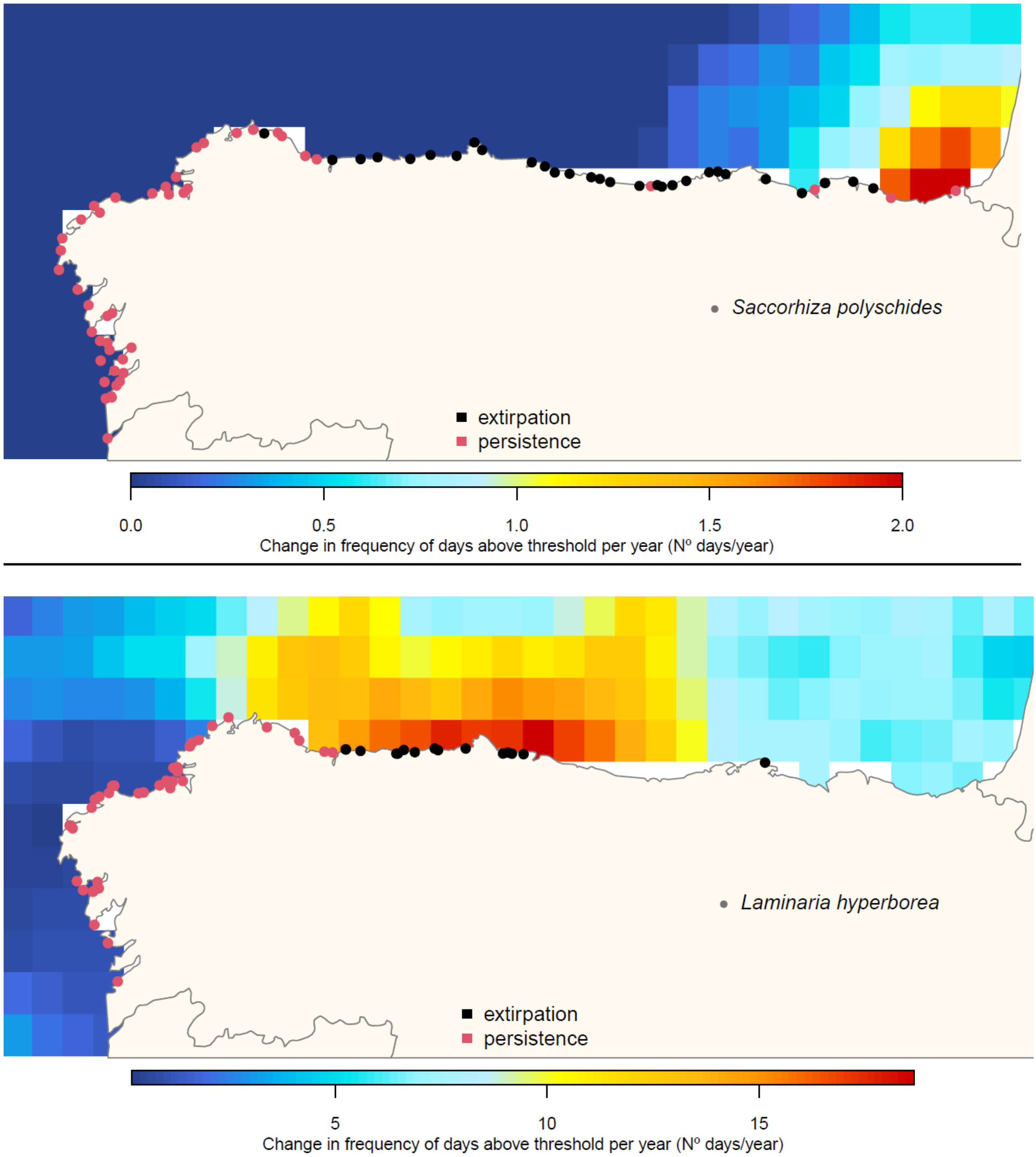

Figure S5 (cont.)

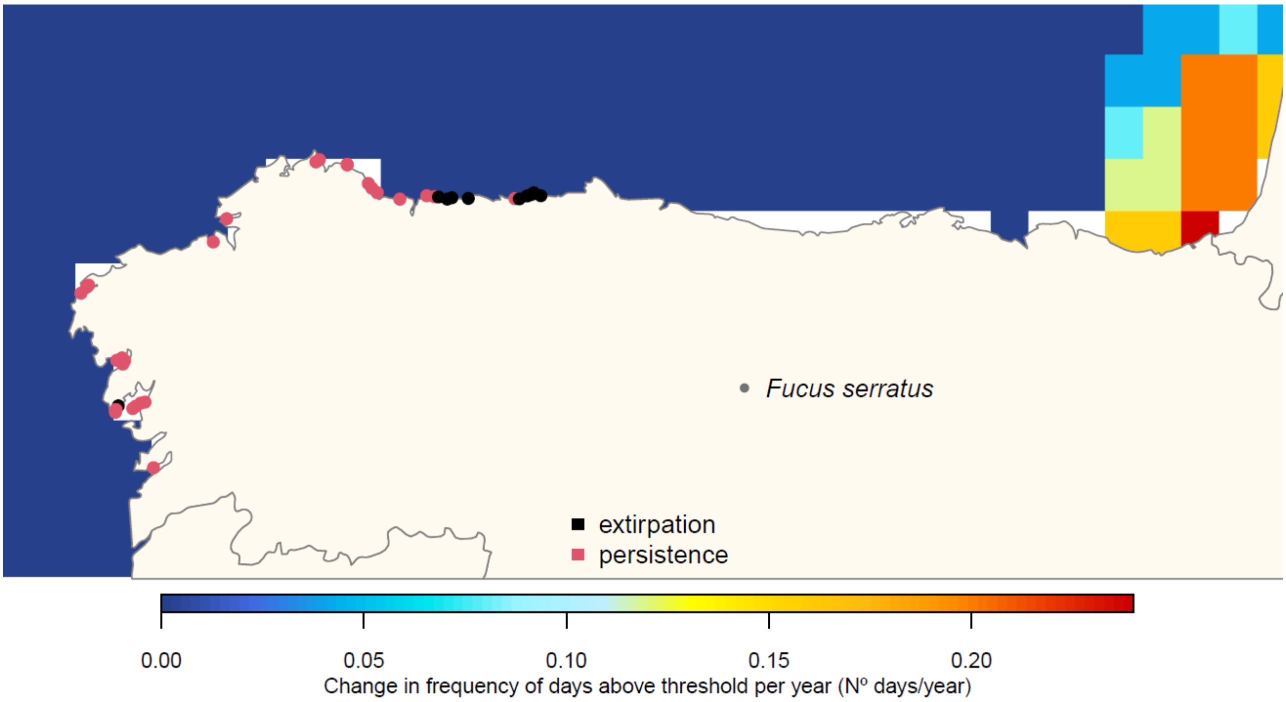

Figure S6

Mean, maximum and minimum SST from January 1982 to December 2015 (encompassing baseline and resurvey periods) at the study area in the Northern Spanish coast. Data were obtained from the NOAA 1/4° daily Optimum Interpolation SST data (daily OISST v2.1 data; Huang et al., 2021). Locally extinct (extirpated) and persistent populations of six macroalgae species were studied (*Fucus serratus, F. vesiculosus, Himanthalia elongata, Laminaria hyperborea, L. ochroleuca and Saccorhiza polyschides*). SST: sea surface temperature.

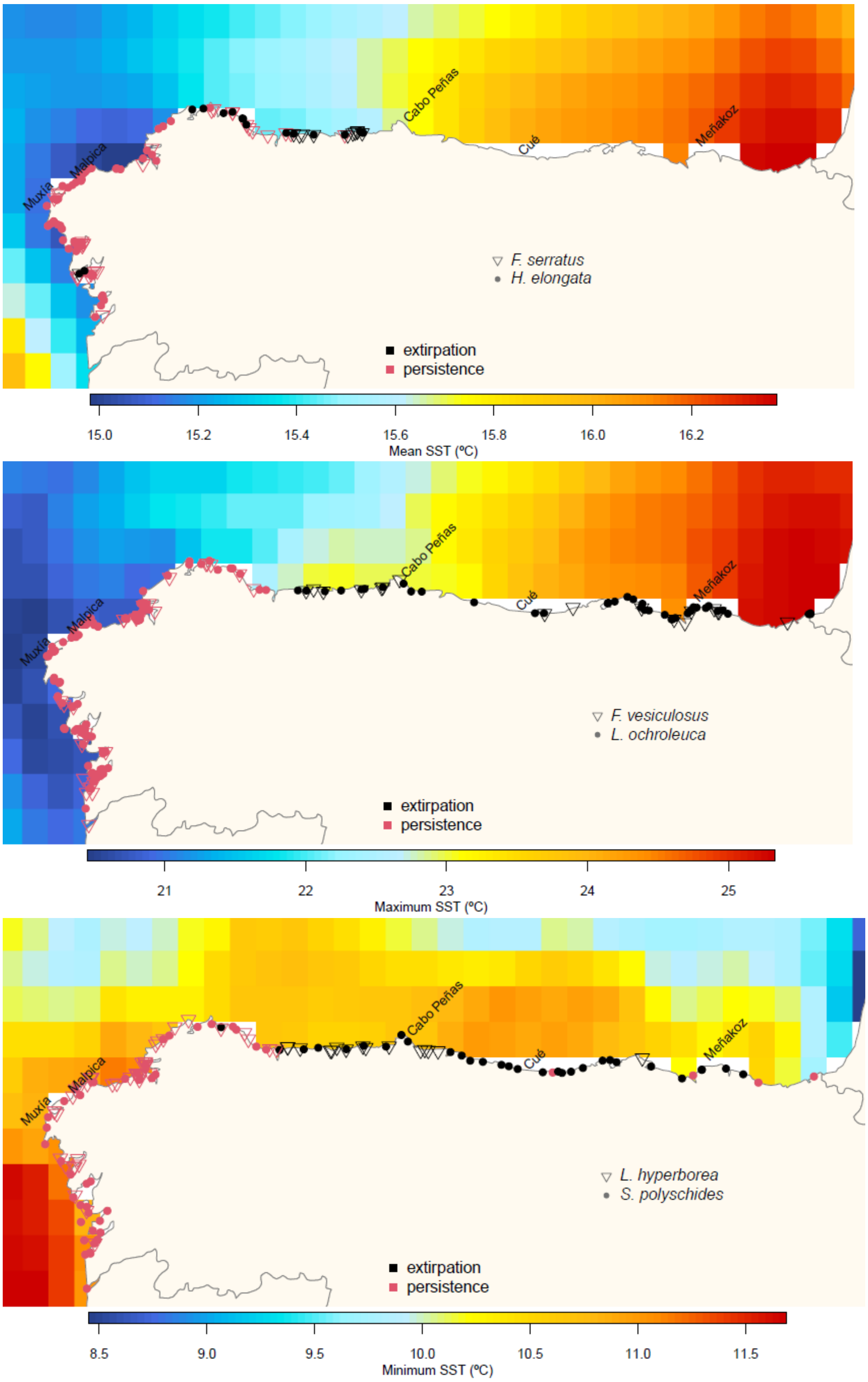

Supplement: Supplementary file 1 — Supplementary Information. [file 41598_2024_64745_MOESM1_ESM.pdf]
